# UNMET COMMUNITY CARE NEEDS AND THE WELL-BEING OF OLDER ADULTS IN CHINA

**DOI:** 10.1093/geroni/igae098.0017

**Published:** 2024-12-31

**Authors:** Shibin Yan

**Affiliations:** Rutgers, The State University of New Jersey, Camden, New Jersey, United States

## Abstract

As China has experienced rapid population aging, the demand for elder care services has increased dramatically. The gap in service demand has become concerning because the traditional provision of eldercare heavily relies on informal care provided by the family. Social and demographic transformations have made the family-reliant care model unsustainable. Community care has become a reasonable and practical option to balance the care demand. However, there is a huge gap between community care provision and need. This prevailing unmet needs in community care services necessitated a deeper understanding of its impact on the well-being of older adults. Using data from the Chinese Longitudinal Healthy Longevity Study, we employ fixed-effect regressions to examine the effects of unmet community care needs on older adults’ health status and subjective well-being (SWB), including specific insights on disabled and childless subpopulations. We find a negative association between unmet needs and both health and SWB, with no significant different effect among subpopulations. Interestingly, Its effect on life satisfaction is moderated by place of residence, with a stronger negative impact in urban areas. These findings highlight the urgent need for more equitable and accessible community care services, particularly for urban older adults, to improve their overall well-being.

